# Complicated Intramural Hematoma with Medionecrosis of the Aortic Wall

**DOI:** 10.1055/s-0041-1729849

**Published:** 2021-10-29

**Authors:** Rosmi P. Thomas, Natalie Gaughan, Daniel Anderson, Stefano Schena

**Affiliations:** 1 Philadelphia College of Osteopathic Medicine, Philadelphia, Pennsylvania; 2 Florida Atlantic University, Boca Raton, Florida; 3 Division of Pathology, The Johns Hopkins Hospital, Baltimore, Maryland; 4 Division of Cardiac Surgery, The Johns Hopkins Hospital, Baltimore, Maryland

**Keywords:** aortic dissection, aortic arch, pathology

## Abstract

Medionecrosis and medial degeneration are rare complications associated with intramural hematomas (IMHs). We present a case of a 69-year-old Asian female with an IMH with medionecrosis and medial degeneration of the aortic wall. The patient underwent successful surgical intervention, and pathological findings were significant for cystic medial degeneration of the aortic wall.

## Introduction

Acute aortic syndromes (AAS) are a spectrum of severe aortic syndromes triggered by a rupture of the vasa vasorum which causes bleeding within the media (intramural hematoma [IMH]) or permits blood to penetrate from the aortic lumen into the media layer (aortic dissection [AD]). 1 Acute AD is the leading cause of 85 to 95% of AAS. 2 AD, a potentially fatal pathology of the aorta, typically presents with a sudden onset of severe thoracic or back pain. Its features are classically confirmed with immediate radiologic imaging. 1 IMHs are a less common subtype of AAS that are due to hemorrhage within the aortic layers in the absence of a primary intimal tear. IMHs have the potential to evolve into a classic AD and/or aortic rupture, and therefore require immediate surgical intervention. 2 3 We, herein, report a case of a patient with an IMH that extends aortic root to the proximal abdominal aorta with significant cystic medial degeneration.

## Case Presentation

**Fig. 1:**
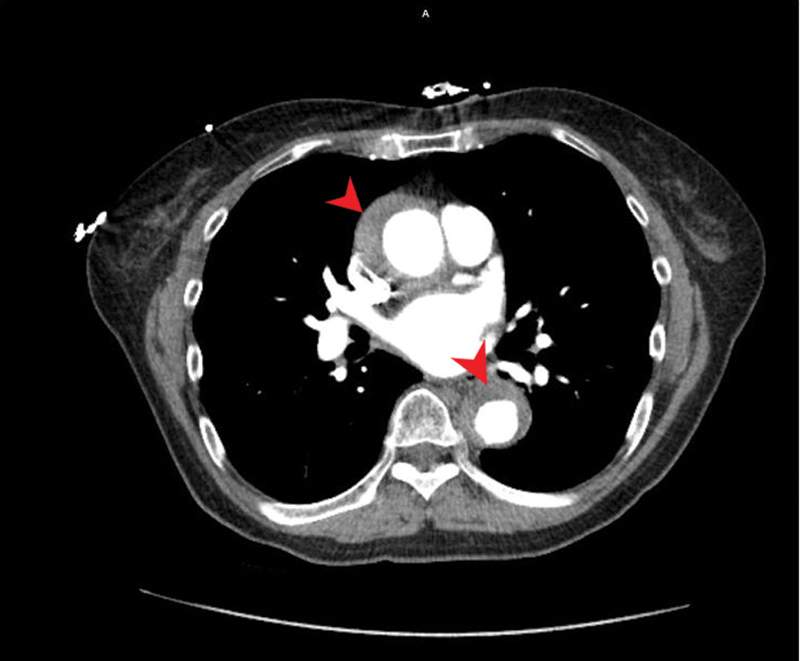
Computed tomography angiogram revealed high dense concentric mural thickening involving the ascending and descending thoracic aorta (arrows) representing an intramural hematoma.

A 69-year-old Asian female presented to a hospital with moderate chest pain that began 3 weeks prior to surgery. She was initially evaluated and managed with antihypertensive and pain medications. However, when the symptoms reoccurred as a new episode of crushing and sharp midthoracic back pain, the patient presented to a different emergency department and was transferred to the Johns Hopkins Hospital. Clinical workup included a chest computed tomography (CT) angiogram that characterized an aorta with diffuse mural thickening. The hematoma was circumferential in nature and measured 8 mm in thickness at the level of the aortic root throughout the ascending aorta, and grew to 15 mm in the descending aorta ( Fig. 1 ). The ascending aorta was also mildly dilated and measured 37 mm in diameter at the level of the pulmonary artery bifurcation. Transthoracic echocardiography (TTE) demonstrated well-preserved left ventricular function with an ejection fraction estimated at 60%, without regional wall motion or valvular abnormalities. Cardiac CT scan with infusion of the coronary arteries suggested the presence of moderate coronary artery disease with no hemodynamically significant stenosis. Based on these findings, a diagnosis of IMH was confirmed, and the patient was advised to undergo urgent surgical repair.

**Fig. 2:**
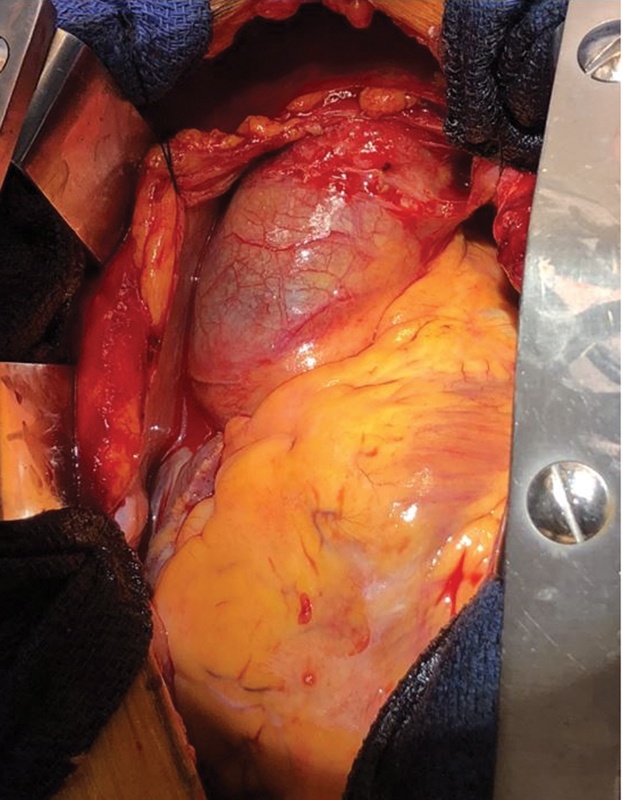
Intraoperative view of bluish discoloration of the adventitia of the ascending aorta.

**Fig. 3:**
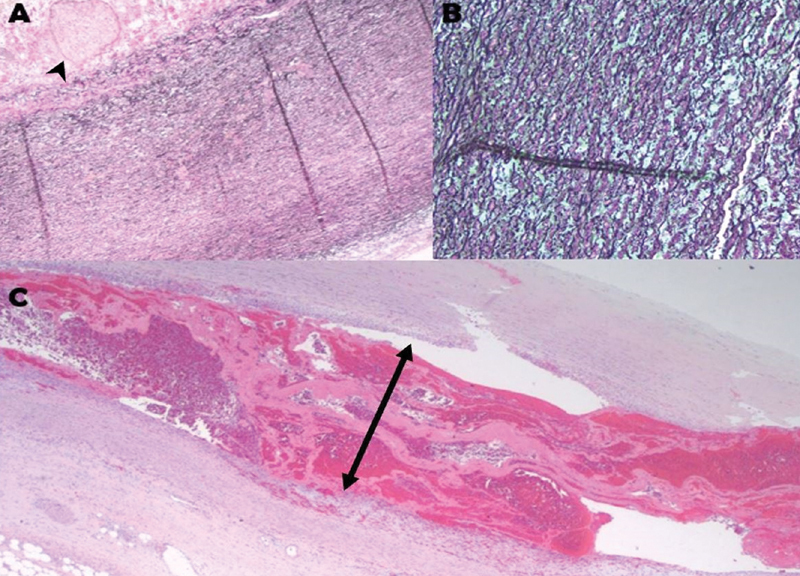
( **A** ) Elastic fibers of the media are shown with Verhoeff–Van Gieson (VVG) stain. Image shows elastic fragmentation with mucopolysaccharide pools (arrowhead) and medionecrosis. ( **B** ) Elastic fiber fragmentation are exhibited with the Movat stain. The image depicts an increased proteoglycan deposition with cyst-like dilations (green background with spaces). ( **C** ) VVG stain of the aortic wall specimen is shown with intima, media, adventitia (from top to bottom) with dissection and entry of blood in the media layer (arrow).

A median sternotomy was performed, and once the pericardium was opened, a moderate bloody effusion was drained. The ascending aorta was visibly affected by an extensive hematoma presenting with bluish discoloration of the adventitia ( Fig. 2 ). The patient was cannulated with the Seldinger technique directly into the distal ascending aorta under TEE guidance to confirm placement into the true aortic lumen. Venous cannulation was bicaval in the superior and inferior vena cava. Deep hypothermic circulatory arrest was performed at 18°C and maintained for 41 minutes. Cerebral protection was performed with retrograde cerebral perfusion at 300 mL/min. Once on cardiopulmonary bypass and following institution of deep hypothermic cardiocirculatory arrest, the proximal ascending aorta was noted to contain a periaortic IMH upon incision. The IMH extended circumferentially through the entire length of the ascending aorta from the noncoronary sinus of the aortic root to the abdominal aorta, dissecting the medial and intimal layers of the vessel ( Fig. 3C ). Inspection of the aortic arch for areas of dissection was performed and demonstrated no additional intimal injury, intimal flaps, or entry sites. Therefore, ascending aorta was replaced entirely to the hemiarch with a 30-mm Hemashield platinum graft. The aortic root layers were recompacted within a sandwich of Teflon strips. Postrepair, intraoperative TEE verified the integrity of the aortic repair with no changes in the aortic valve's preserved continence. The patient's postoperative course was unremarkable, and she was discharged home on postoperative day 5. The descending aorta will be monitored with yearly CT angiography.

Postoperative pathology findings revealed cystic medial degeneration of the aortic wall, characterized by elastic fiber fragmentation with the formation of mucopolysaccharide pools and medionecrosis, defined as an apparent loss of nuclei in the media ( Fig. 3A ). Blood was also noted to split the medial and intimal layers of the aorta ( Fig. 3B ).

## Discussion

IMH has an incidence of 1.2 per 100,000, making it the rarest form of AAS. Various complications can arise with IMH, such as pericardial effusions, pleural effusion, and the more serious complication of progression to an AD. 1 The clinical presentation of IMHs is similar to that of an AD; however, due to the segmental nature of IMH, radiation of pain to the head or lower extremities is less common. 4 Traditionally, ascending aortic hematomas (Type A) present with chest pain, while descending aortic hematomas (Type B) manifest with upper or lower back pain. 3 The presentation within this case of a Type A IMH with upper back pain highlights the importance of imaging in diagnosing an IMH. 1

Despite the physiologic difference in Type A IMH and ADs, there is no significant difference in in-hospital mortality between the two pathologies. 5 In a study from the International Registry of Acute Aortic Dissection (IRAD), it was found that those diagnosed with an aortic IMH were likely to progress to rupture 20% of the time. 2 Medical management of a Type A aortic IMH was shown to have a 40% mortality rate; while surgical intervention showed increased survivability, with a mortality rate of 24.1%. 5 The deaths associated with medically managed Type A IMH patients were related to aortic rupture, indicating that immediate surgical intervention is critical in cases indicated as a “severe” IMH. 1 5

In the presented case, medionecrosis of the medial layer highlights an atypical complication associated with IMH. The aorta is a large elastic artery with the medial layer constituting its greatest component. 6 Medionecrosis, defined as loss of smooth muscle cell nuclei in the medial aortic layer, was indicated on histopathology. Laminar medial collapse, a compaction of medial elastic fibers that create thinning of a lamellar unit secondary to smooth muscle cell loss, is a rarely reported complication associated with IMH. 6 In addition, medial degeneration, defined as a formation of pools of mucopolysaccharides that contribute to smooth muscle disorganization, was noted. There are various aortic diseases that may present with cystic medial necrosis such as aortic aneurysm associated with bicuspid aortic valve, aortic aneurysm associated with cocaine use, coarctation of the aorta, and the diverticulum of Kommerell. 6 However, the combination of medionecrosis, laminar medial collapse, and smooth muscle disorganization may be associated with a progressing weakness in the aortic wall that can lead to AAS.

IMH with associated medionecrosis and medial degeneration is a type of AAS with uncommon occurrence. The ability for complicated IMHs to progress to an AD emphasizes the importance of early recognition of varied clinical presentations. 1 Rapid surgical intervention in cases of severe IMH, such as the one implemented in the patient presented, is crucial to patient survival and management. 6
